# Real-Time 2-D Lidar Odometry Based on ICP

**DOI:** 10.3390/s21217162

**Published:** 2021-10-29

**Authors:** Fuxing Li, Shenglan Liu, Xuedong Zhao, Liyan Zhang

**Affiliations:** College of Mechanical & Electrical Engineering, Nanjing University of Aeronautics and Astronautics, Nanjing 210016, China; lfx_mail@163.com (F.L.); cnc@nuaa.edu.cn (X.Z.); zhangly@nuaa.edu.cn (L.Z.)

**Keywords:** 2-D lidar, multi-scale, feature extraction, motion estimation

## Abstract

This study presents a 2-D lidar odometry based on an ICP (iterative closest point) variant used in a simple and straightforward platform that achieves real-time and low-drift performance. With a designated multi-scale feature extraction procedure, the lidar cloud information can be utilized at multiple levels and the speed of data association can be accelerated according to the multi-scale data structure, thereby achieving robust feature extraction and fast scan-matching algorithms. First, on a large scale, the lidar point cloud data are classified according to the curvature into two parts: smooth collection and rough collection. Then, on a small scale, noise and unstable points in the smooth or rough collection are filtered, and edge points and corner points are extracted. Then, the proposed tangent-vector-pairs based on edge and corner points are applied to evaluate the rotation term, which is significant for producing a stable solution in motion estimation. We compare our performance with two excellent open-source SLAM algorithms, Cartographer and Hector SLAM, using collected and open-access datasets in structured indoor environments. The results indicate that our method can achieve better accuracy.

## 1. Introduction

Simultaneous localization and mapping (SLAM) technology has developed rapidly and is widely used. Whether in automated driving [1,2,3] or unmanned industrial transportation, or service robots, SLAM covers most scenarios from high-speed to low-speed motion. The field of service robots mainly includes security robots, guide robots, sweeping household robots, etc. The application scenarios range from a broad outdoor environment to a limited indoor environment. Localization is the essential task to be completed in the motion estimation module in SLAM [4]. Although various sensors are used for motion estimation, such as IMU, GNSS, and camera [5], lidar is still highly competitive in SLAM applications, thanks to its high measurement accuracy, high anti-interference ability, and stability to light illumination.

This paper studies the localization of service robots using 2-D lidar in indoor environments. For lidar SLAM, filtering-based algorithms were first widely used and applied to 2-D lidar [6]. The most likely pose of a robot is estimated with the distribution of sequential sensor data. Such techniques are usually sensitive to computing resources. With the progress of nonlinear solvers, lidar SLAM gradually adopts optimization methods to solve motion estimation, such as KartoSLAM [7], HectorSLAM [8], LagoSLAM [9], and Cartography [10]. These methods do not deliberately extract features when performing data association but use raw data to perform motion estimation directly. In 3-D lidar SLAM, downsampling and feature extraction are performed in the data preprocessing stage to obtain less noisy and more stable data. This difference in data processing is also due to the fact that 3-D lidar produces more information than 2-D lidar, so it is objectively more difficult to extract effective and rich features from 2-D lidar data. Moreover, a general single-scale feature extraction procedure can easily lead to information loss.

For localization tasks in indoor structured environments, 2-D lidar SLAM is sufficient to solve most problems; however, minimal attention has been given to multi-scale feature extraction of the front-end of 2-D lidar SLAM. Therefore, this paper focuses on a 2-D lidar-based approach.

## 2. Related Work

The essence of the localization task is to match the sequential sensor data, known as scan-matching, which can be solved by the optimization-based method. The method usually forms a graph model with vertices and edges, in which the vertices are the variables to be optimized, and the edges are the constraint relationship between the vertices [11], and then the model can be transformed into a least-squares problem.

ICP [12] and its variants [13,14,15,16] can be used to match lidar point clouds between different frames. These techniques vary from point-to-point to point-to-plane in data correspondence, and some remove obvious outliers with geometric features, such as normal vector and curvature. Some of them are specially designed for 3-D data. For example, GICP [14] uses the correspondence between points and planes, and NICP [15] applies the 3-D structure around the points to form data association. In 2-D lidar data, it is difficult to find and utilize geometric features, such as a plane or a normal vector to a plane. Moreover, it is time-consuming for the process of finding the closest points. Tian et al. [17] proposed a method for the acceleration of data correspondence with an assisted intensity to reduce the computational cost and avoid divergences.

Apart from scan-matching, some methods focus on the distribution of lidar point clouds in the environment to estimate the pose of a robot. For example, Gmapping [18] and CoreSLAM [19] both utilize improved particle filters for localization, but they are sensitive to computing resources or have poor positioning performance. Ren et al. [20] proposed an improved correlative scan matching in a probabilistic framework, which can evaluate a robot’s pose with the distribution of lidar data.

LagoSLAM [9] and KartoSLAM [7] construct an SLAM problem with a pose graph model, which finds what pose state the robot is in that is most likely to obtain the current observations. HectorSLAM [8] and Cartographer [10] match the point cloud with the map. The scan matcher also needs to find the most likely position of the laser point on the map to retrieve the pose of a robot. The improved HectorSLAM and a control network constraint-based method [21] focus on reducing the cumulative error in the backend [22].

These scan-matching methods or pose estimation techniques designed for 2-D SLAM usually do not specially extract features for 2-D lidar data; some non-essential data are retained when processing lidar data. While feature extraction is common in 3-D lidar SLAM, it is a crucial part of the front end in LOAM [23] and LeGO-LOAM [24]. LOAM extracts edge points and plane points and uses the distance from the point to line and point to plane for data association. LeGO-LOAM segments the point cloud in advance before feature extraction, which greatly reduces noise and improves the efficiency of feature extraction. Grant et al. [25] proposed a new planar feature extraction method for fast point cloud registration. When faced with a large number of dense clouds, one can apply bundle adjustment to lidar SLAM to handle large-scale edge and plane features [26] or use sliding windows to increase the speed of the system [27].

These feature extraction procedures only extract features on a single scale, and the information in the point cloud is not taken into account sufficiently, such as PCA-based line detection [28]. One of the advantages of multi-scale feature extraction is that various levels of information can be retrieved. The construction of geometric features is usually very sensitive to the size of the neighborhood, and the size of the scale is not easy to control. Conversely, the data structure of multi-scale features can be used to construct an effective search space or to omit the secondary search process, which greatly accelerates the speed of data association. Pfister et al. [29] used 2-D lidar data to construct multi-scale point and line features and adopted a coarse-to-fine strategy to traverse matching points, significantly reducing the search space for fine-scale features and improving the robustness of scan-matching. Wu et al. [30] extracted the multi-scale corner features with curvature detector in 2-D lidar data. To better classify the laser point cloud, Hang et al. [31] extracted multi-scale features and embedded them in a low-dimensional and robust subspace to obtain a more compact geometric feature representation and achieve a better classification performance. Bosse et al. [32] designed a feature descriptor that captured the local structure, and the descriptor is robust to measurement noise.

This paper considers the influence of noise on sensor data, applies multi-scale metrics to achieve multi-level and robust feature extraction, and then combines with the ICP and the proposed tangent-vector pair to obtain robust motion estimation. Due to the multi-scale data structure, searching and relating data can be carried out quickly, which can save time in the iterative solution process.

Naturally, during the movement of lidar, one inherent problem is point cloud distortion. It takes time for lidar to transmit and receive signals, which results in the beams inevitably being received at different positions during the lidar movement. Moreover, the more intense the movement, the more pronounced the change. This phenomenon is known as motion distortion. Generally, auxiliary sensors are required to calculate the distorted movement of each point by linear interpolation and remove the distortion. Another common way is to apply the extended Kalman filter (EKF) to integrate lidar, IMU, and other information [33,34] to the de-skew cloud. However, when the sensor movement is not so intense, it is acceptable to ignore this movement distortion. Or, it can be assumed that the sensor moves at a constant speed to calculate the movement of each point. Specifically, in our case, the lidar moves at a low speed of 0.3–0.5 m/s, so we ignore the distortion.

## 3. System Overview

### 3.1. Lidar Hardware

**Figure 1:**
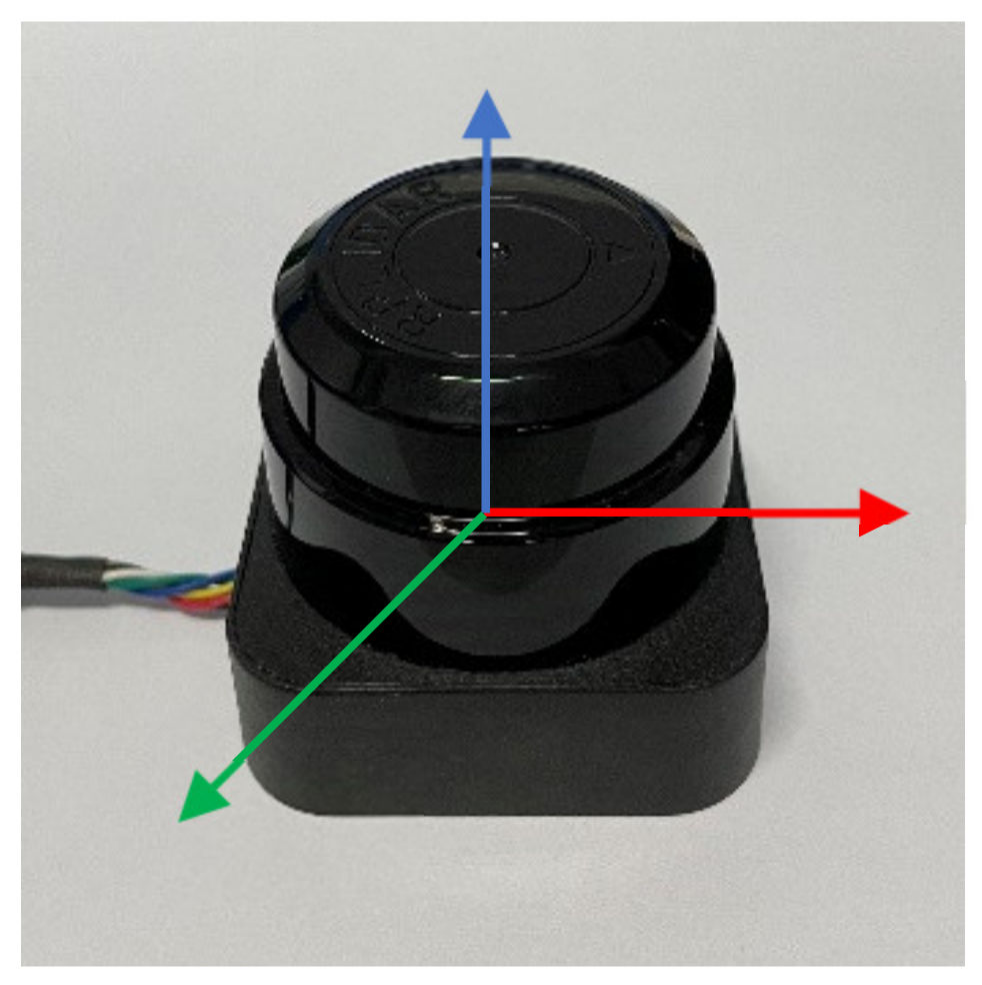
Single-line mechanical rotating lidar.

As shown in Figure 1, this paper utilizes an RPLIDAR S1 manufactured by SLAMTEC. The lidar has a horizontal field of view of 360 degrees, a measurement distance of 0.1–40 m, a scanning frequency of 10 Hz, a measurement resolution of 3 cm, and a measurement accuracy of ±5 cm. All obtained point clouds are in the lidar coordinate {*L*} of the left hand.

### 3.2. Software System Overview

**Figure 2:**
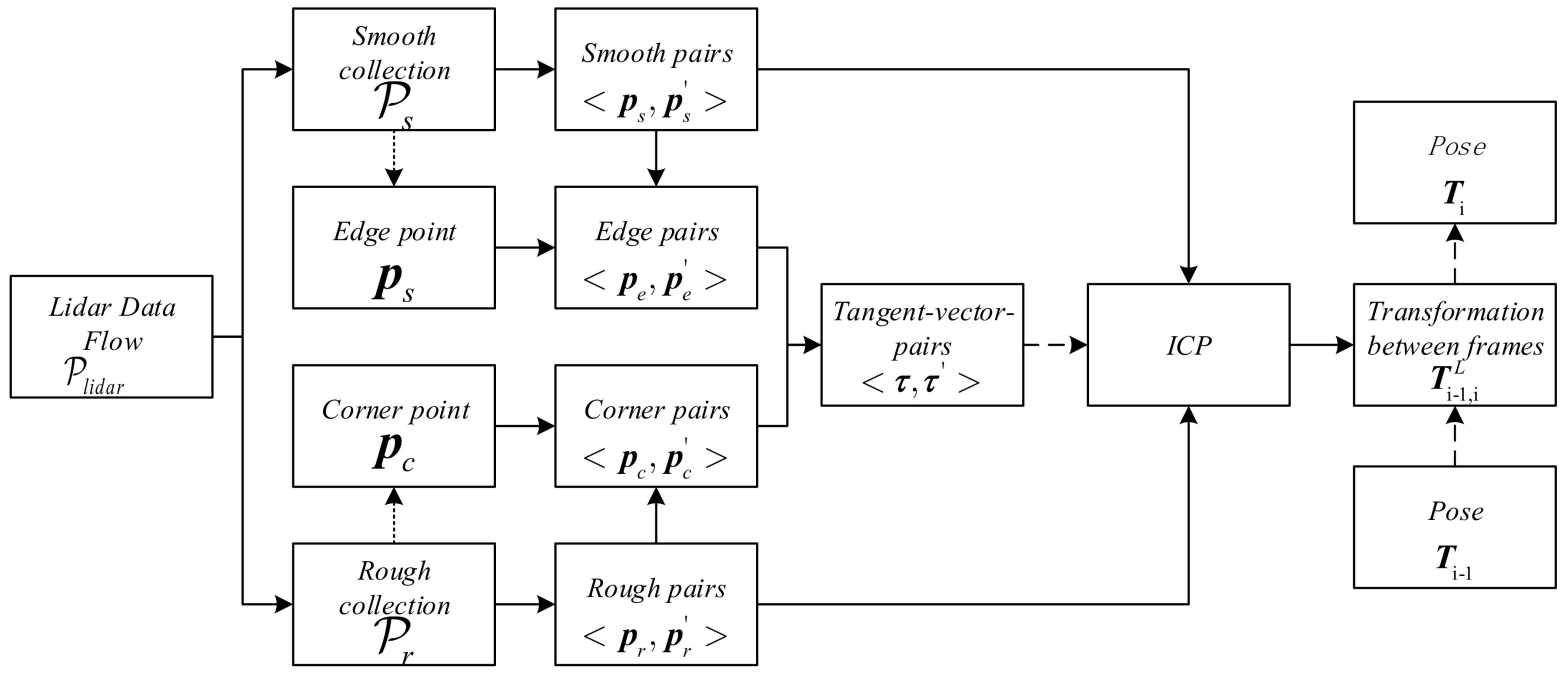
i represents the current frame, and i−1 represents the previous frame. ${Τ}_{i-1,i}^{L}$ is the transformation matrix between frame i and i−1 in the coordinate system {*L*}.

Downsampling raw data and eliminating outliers can reduce the burden of computational resources and improve the real-time performance of the system; therefore, this paper designs a multi-scale feature extraction procedure that can reserve the appropriate data. In Figure 2, we first classify the lidar data into two parts, smooth collection $P_{s}$ and rough collection $P_{r}$, according to their curvature defined in Formula (1). Then, the corresponding point pairs $<p_{s},p_{s}^{\prime }>$ and $<p_{r},p_{r}^{\prime }>$ are obtained between the sequential sensor data. Respectively, the edge point $p_{e}$ and corner point $p_{c}$ are extracted from the smooth collection and rough collection according to the constraint conditions we proposed in Formulas (3) and (4). In addition, the point pairs $<p_{e},p_{e}^{\prime }>$ and $<p_{c},p_{c}^{\prime }>$ are obtained, and they yield the tangent-vector pairs $<\tau ,\tau^{\prime }>$. The motion estimation problem is solved by our ICP variant algorithm proposed in Formula (6). Then, the transformation between two lidar frames can be obtained, and the current lidar pose-state in the world coordinate is established.

## 4. Lidar Odometry

### 4.1. Feature Extraction

Central to our method is a multi-scale metric procedure that allows feature analysis at multiple scales, using the local neighborhood’s size as a scale parameter.

On a large scale, we evaluate the smoothness of a point using a neighborhood composed of more points. The calculation process similar to the method in [20] is Formula (1) and we set $\left|C\right|$ to 10. When $\kappa_{1}>0.1$, a point is classified to rough collection; otherwise, it is classified to smooth collection. However, the neighborhood’s rough points or sensor noise can easily affect the actual smooth point, which is detected as a rough point. Although, when having a large sample size in the calculation, a few misclassified points will not significantly affect the final result. For example, when we use the ICP method, we will not deliberately exclude such points because enough correct correspondence pair samples are involved in the evaluation:

(1) $$\kappa_{1}=\frac{1}{\left|C\right|}\left\|\sum_{j\in C,j\neq k}\left(p_{j}-p_{k}\right)\right\|$$

When the total sample size becomes smaller, such misclassified points have a more significant impact. We must consider the data on a smaller scale to utilize two adjacent points to compute the tangent vector in Formula (2) and then define the straightness similar to the method of [35] in Formula (3) to evaluate the smoothness of a point in a small area. Moreover, we use the tangent vector difference in Formula (4) to evaluate the extent of noise influence on points because the sensor has the measurement accuracy, for example, the lidar measurement accuracy we use is ±5 cm. When the lidar scans a plane, we clearly notice that the measuring point is throbbing in a specific range, and this type of point is easily recognized as a rough point, but it belongs to a point on a straight line. Therefore, we implement the small-scale extraction for the points classified by the large-scale feature extraction, and the filtered points are used for rotation estimation in Section 4.2.

In Formula (2), $\tau_{k}$ is the tangent vector of the *k*-th point $p_{k}$. In Formula (3), $l_{k}$ is the straightness of the *k*-th point $p_{k}$. $C_{k}$ is a collection of the neighborhood and $\left|C_{k}\right|$ is set to two. In Formula (4), $\kappa_{2}$ represents the difference between two sequential tangent vectors:

(2) $$\tau_{k}=\frac{p_{k+1}-p_{k}}{\left\|p_{k+1}-p_{k}\right\|}$$

(3) $$l_{k}=\tau_{k}\cdot \frac{1}{\left|C_{k}\right|}\sum_{j\in C_{k},j\neq k}\left(p_{j}-p_{k}\right)$$

(4) $$\kappa_{2}=\frac{∠\tau_{k-1}\tau_{k}}{\left\|\frac{p_{k+1}-p_{k}}{2}\right\|+\left\|\frac{p_{k}-p_{k-1}}{2}\right\|}$$

**Figure 3:**
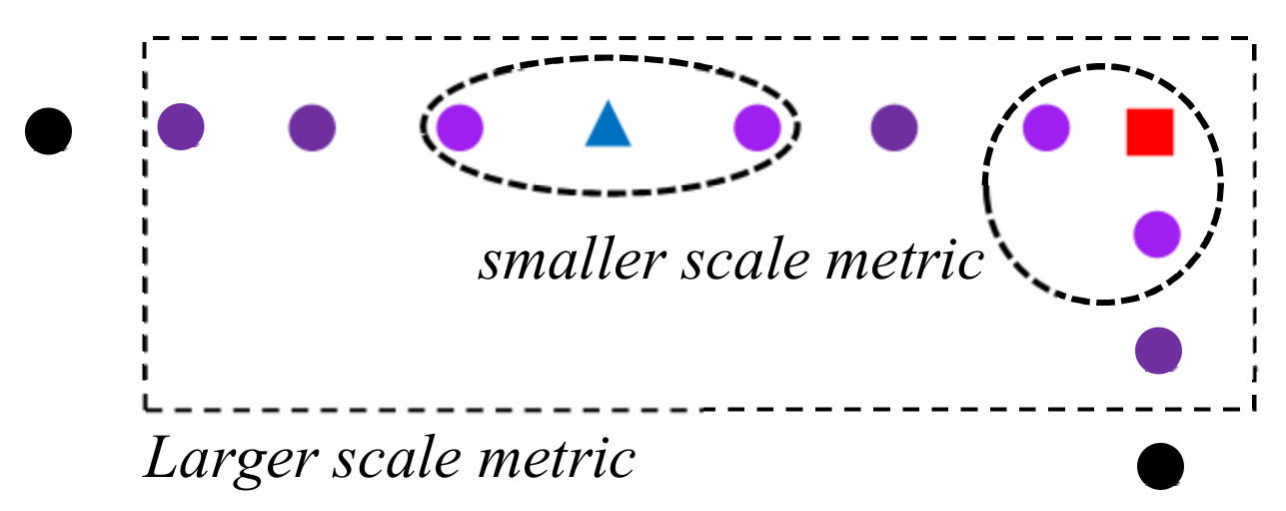
The blue triangle is an edge point, and the red square is a corner point. When classified at a large scale, the edge point is likely to be classified into corners.

**Figure 4:**
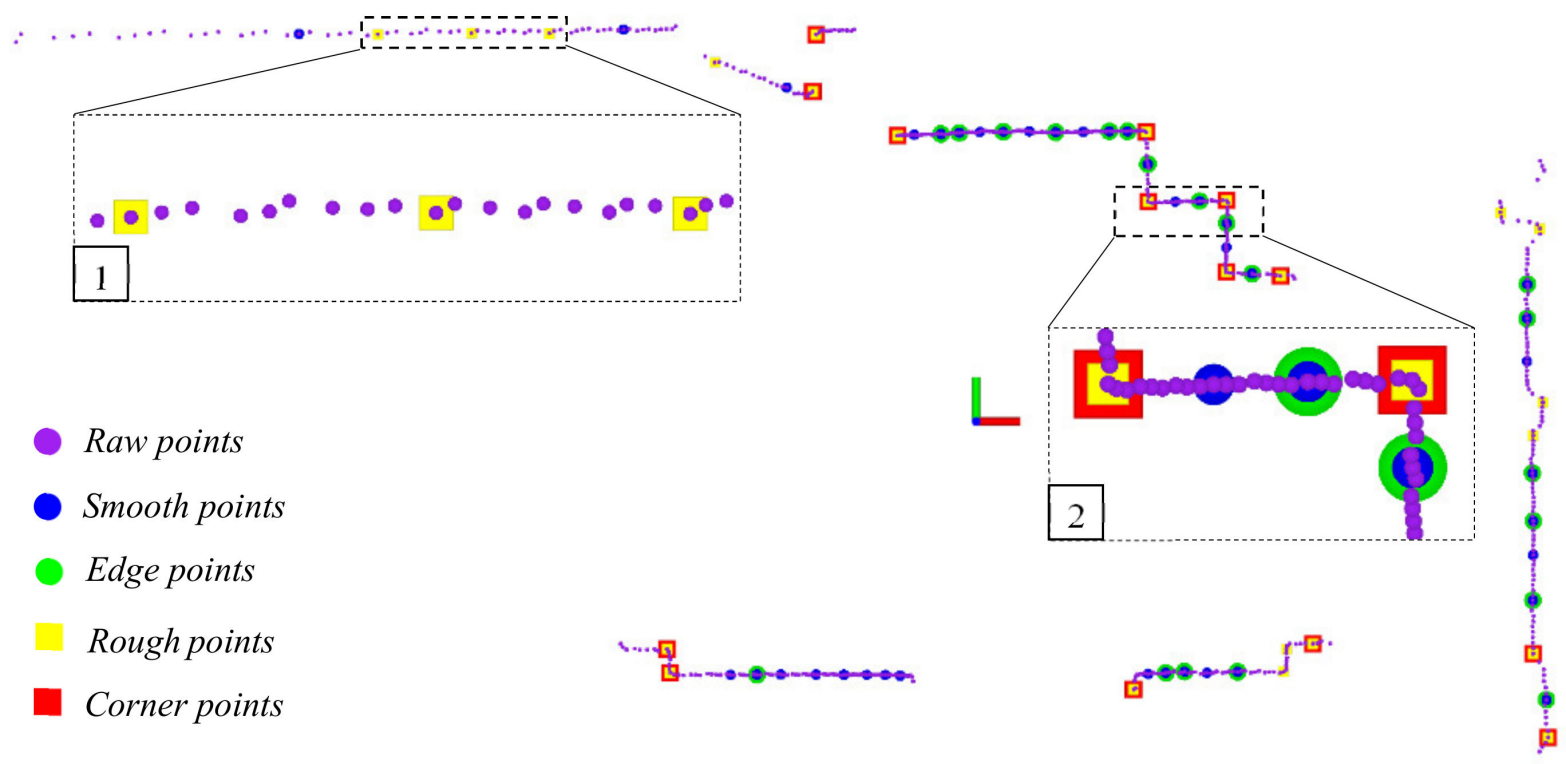
The result of the multi-scale metric procedure. In dotted box 1, the raw points are not strictly distributed in a straight line due to the influence of noise, and we find large-scale metric results in misclassified points. The yellow rectangle should not belong to rough points. In dotted box 2, the red corner points and green edge points are in the correct category.

Generally, in the smooth collection, when the straightness of a point is very large ($l_{k}>1.5$), the *k*-th point and its neighbors should be on a straight line. If the influence of the noise is small ($\kappa_{2}<0.5$), the point should be classified as an edge point; in the rough collection, when the straightness of a point is very small ($l_{k}<1$), it may be at corners. If the “noise” is considerable ($\kappa_{2}>1$), it means that the tangent vector changes significantly. The point should be classified as corner points. In this way, we have a small number of more stable point sets by small-scale classification. Figure 3 illustrates a schematic diagram of the different results produced by small-scale and large-scale neighborhoods. Multi-scale classification can exclude some single-scale misclassification points. A specific example is shown in Figure 4.

### 4.2. Motion Estimation

The raw data are classified into four categories: smooth collection, rough collection, edge point, and corner point. The large-scale metric classified data, smooth collection and rough collection, are used as the input of the ICP method. In ICP, the KD-tree is used to construct a search space to find the relevant point, that is, the nearest point, and then iterated until convergence or the maximum number of iterations is reached. During the iteration, the method first finds the closest point and then generates a preliminary related point pair, and Formula (5) must be met; otherwise, the point pair is discarded:

(5) $$\left\|\kappa_{1}-\kappa_{1}^{\prime }\right\|<\varepsilon_{\kappa_{1}}$$

In our experimental setup, we set $\varepsilon_{\kappa_{1}}$ to 0.005. Then, all the point pairs $<p_{s},p_{s}^{\prime }>$ and $<p_{r},p_{r}^{\prime }>$ build up the set P. We use $M_{1}\in SE(3)$ to represent the transformation matrix and the corresponding Lie algebra is ξ∈se(3). The formula of ICP is written in the form:

(6) r1P,ξ=pn−expξ^pn′

where expξ^→M1 is the mapping from Lie algebra to Lie group, and $<p_{n},p_{n}^{\prime }>$ is the pair in P.

For small-scale classified point cloud data, edge points and corner points are collected from the points extracted from large-scale features, and the relationship between points is built. However, all point pairs must meet the conditions of the following Formulas (3) and (4) or are discarded:

(7) $$\left\|\kappa_{2}-\kappa_{2}^{\prime }\right\|<\varepsilon_{\kappa_{2}}$$

(8) $$\left\|l_{k}-l_{k}^{\prime }\right\|<\varepsilon_{l_{k}}$$

**Figure 5:**
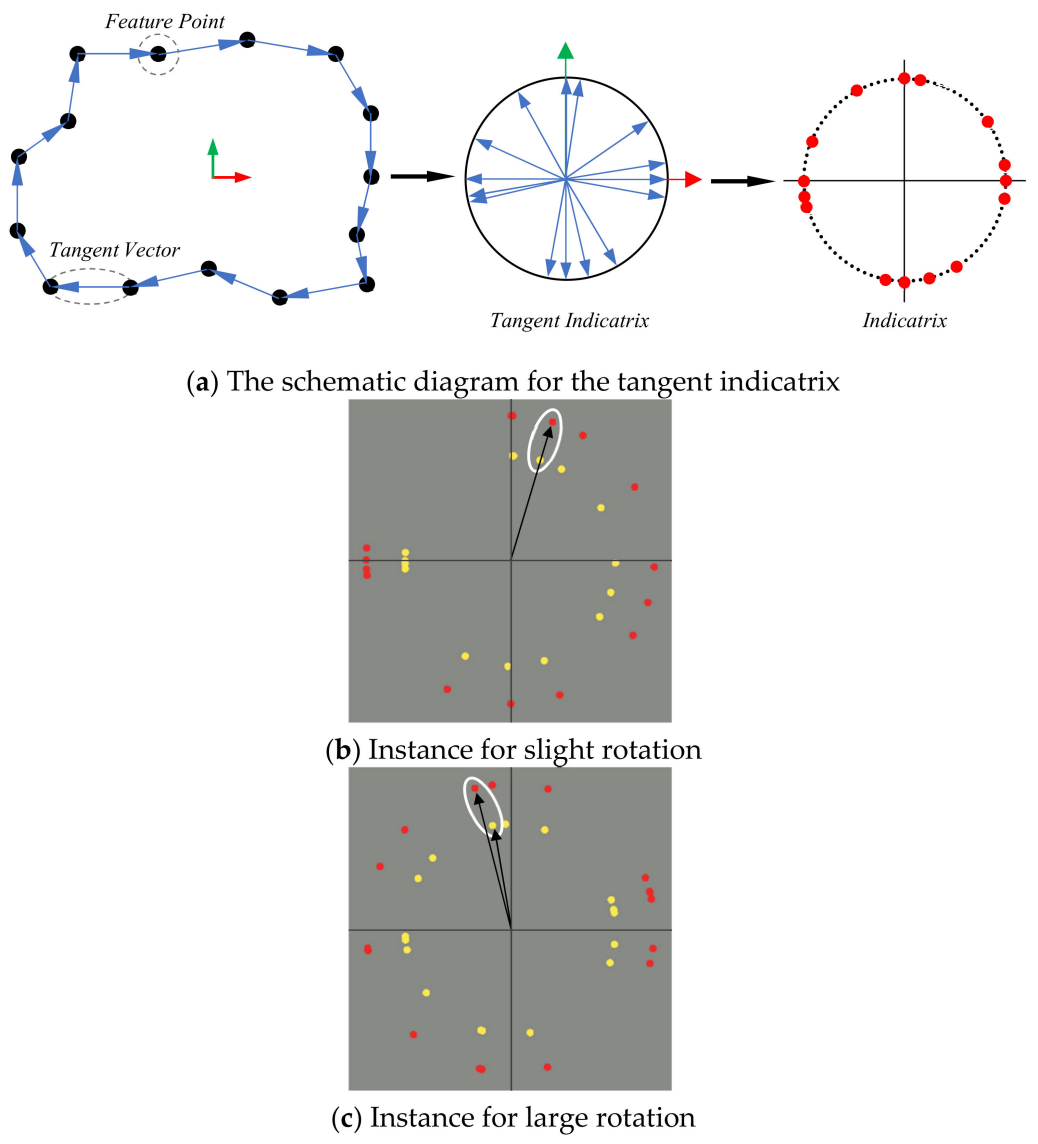
The illustration of the tangent-vector pair for steadily solving rotation DoF. (**a**) Left shows an example when the tangent vector is computed by two points, so the tangent vector is end-to-end. (**b**) The tangent-vector pair is in the white ellipse, the angular deviation of the vector pair is 0.63 degrees, and the average angle deviation of all the vector pairs is 0.98 degrees. The sensor is almost not rotating. (**c**) In the white ellipse, the angular deviation of the vector pair is 4.52 degrees, and the average angle deviation of all vector pairs is 3.80 degrees. The sensor is rotating.

In practice, $\varepsilon_{\kappa_{2}}$ and $\varepsilon_{l_{k}}$ are set to 0.05. However, this paper does not directly use edge points and corner points but uses these points to calculate the tangent-vector pair to estimate the degree of freedom of rotation. First, we explain how to use the tangent-vector pair to calculate the rotation angle. In the left of Figure 5a, there are several feature points, which can be edge points or corner points, and then their tangent vectors are calculated. In this paper, these tangent vectors are connected end to end. These feature points are stable, so the shape of the formed polygon is also stable, which is why the points generated by the large-scale classification method are not used. It can be assumed that a polygon rotates slightly on the plane. If we know the rotation angle of each side, then the rotation angle of the polygon can be easily calculated. Here, the sides of the polygon are the tangent vectors. For the convenience of presentation, all unit tangent vectors are moved to the origin of the coordinate system to form a tangent indicatrix, which can abstractly represent the distribution state of polygon sides, and further omit line segments and simplify into an indicatrix. In practice, the shape of the polygon represented by the indicatrix extracted from the previous frame and the indicatrix of the current frame should be basically the same, but the rotation angle is different, which is why the tangent-vector pair can estimate the degree of freedom of rotation. A specific example is shown in Figure 5b,c.

For the rotation matrix R2∈SO(3),expϕ^→R2, and the relative transformation matrix $M_{2}\in SE(3)$ in Formula (9). The corresponding tangent vector pair is $<\tau_{k},\tau_{k}^{\prime }>$. The solution for rotation of tangent-vector pairs is in Formula (10) and $<\tau_{m},\tau_{m}^{\prime }>\in T$:

(9) $$M_{2}=\left[\begin{array}{cc} R_{2} & t_{2}\\ 0^{⊤} & 1 \end{array}\right],t_{2}=0,M_{2}\in SE(3)$$

(10) r2T,ϕ=τm−expϕ^τm′

The full motion estimation solution is in Formula (11), where ρ is the Cauchy kernel function. The Lie algebra perturbation model is used to solve the Jacobian matrix of two error terms Formulas (6) and (10):

(11) $$\underset{\xi ,\phi }{min}\left\{\sum_{}^{N}{\left\|r_{1}\left(P,\xi \right)\right\|}^{2}\right.+\left.\sum_{}^{M}\rho {\left\|r_{2}\left(T,\phi \right)\right\|}^{2}\right\}$$

(12) J1=∂r1∂δξ=−expξ^pn′⊙

(13) J2=∂r2∂φ=expϕ^τm′^

where δξ and φ are small perturbations of ξ and ϕ, respectively. We obtain the transformation between frames in Formula (14). s is the number of edges and corner pairs. ε is a threshold and set to 50:

(14) $$T^{L}=M_{1}⊗M_{2}≜\left[\begin{array}{cc} R_{1} & t_{1}\\ 0^{⊤} & 1 \end{array}\right]⊗\left[\begin{array}{cc} R_{2} & t_{2}\\ 0^{⊤} & 1 \end{array}\right]=\left[\begin{array}{cc} R & t_{1}\\ 0^{⊤} & 1 \end{array}\right],R=\left\{\begin{array}{c} R_{1},s<\varepsilon \\ R_{2},s\geq \varepsilon \end{array}\right.$$

Therefore, the problem of motion estimation can be solved by the L-M method in Formula (15):

(15) $$T^{L}\leftarrow T^{L}-{\left(J^{⊤}J+\lambda D^{⊤}D\right)}^{-1}J^{⊤}r\left(\cdot \right)$$

where D is a non-negative diagonal matrix, which is taken as the square root of the diagonal elements of $J^{⊤}J$. λ is a parameter defined in the L-M method. After evaluating the motion between frames in the lidar coordinate, we obtain the relationship of the sensor’s ego-motion in the world coordinate in Formula (16). The motion estimation algorithm is shown in Algorithm 1. Finally, continuous motion estimation constitutes a 2-D lidar odometry.

(16) $${Τ}_{i}={Τ}_{i-1,i}^{L}{Τ}_{i-1}$$

**Algorithm 1:** Motion Estimation**Input:** smooth collection $P_{s}$, rough collection $P_{r}$
**Output:** transformation matrix $T^{L}$
1:  set $T^{L}$ to the identity matrix2:  **for** *i* = 0 to *max iteration* **do**3:   **for** each smooth point $p_{s}$ in $P_{s}$ or rough point $p_{r}$ in $P_{r}$
**do**4:    Find the closest point $p_{s}^{\prime }$ and $p_{r}^{\prime }$ for $p_{s}$ and $p_{r}$ in last scan regarding
    to smooth collection and rough collection, respectively.5:    All the point pairs $<p_{s},p_{s}^{\prime }>$ and $<p_{r},p_{r}^{\prime }>$ yield to Formula (5)6:   **end for**7:   Compute $\tau_{k},l_{k},\kappa_{2}$ for all points in $<p_{s},p_{s}^{\prime }>$ and $<p_{r},p_{r}^{\prime }>$.8:   **for** each point $p_{s}$ in $<p_{s},p_{s}^{\prime }>$ and $p_{r}$ in $<p_{r},p_{r}^{\prime }>$
**do**9:    Classify point as edge $p_{e}$ when $l_{k}>1.5$ and $\kappa_{2}<0.5$,10:    Classify point as corner $p_{c}$ when $l_{k}<1$ and $\kappa_{2}>1$.11:    All the point pairs $<p_{e},p_{e}^{\prime }>$ and $<p_{c},p_{c}^{\prime }>$ yield to Formulas (7) and (8).12:   **end for**13:   Compute new $\tau_{k}$ for all the points in $<p_{e},p_{e}^{\prime }>$ and $<p_{c},p_{c}^{\prime }>$,
   then obtain $<\tau ,\tau^{\prime }>$.14:   Use $<p_{s},p_{s}^{\prime }>$ and $<p_{r},p_{r}^{\prime }>$ as input of Formula (6).15:   Use $<\tau ,\tau^{\prime }>$ as input of Formula (10).16:   Update $T^{L}$ for next iteration.17:   **if** the convergence is satisfied **then**18:    Return $T^{L}$
19:   **end if**20:  **end for**

## 5. Experiment

We performed algorithm verification in an Ubuntu system (18.04 LTS) running Robot Operation System (ROS in Melodic version) on an Intel 3.7 GHz, 6-core CPU, 16 GiB memory computer. The algorithm uses no more than two cores and no more than 2 GiB memory.

### 5.1. Our Datasets

We collected three datasets in different areas of the underground garage and one dataset in our lab. We used tape to plan the trajectory points every 2.5 m and then held the lidar to walk clockwise along with the trajectory points in straight lines. In the underground garage, the lidar was above the car’s roof all the way to ensure that the collected data were all relative to walls and pillars. In the lab, people were walking around. The starting point and ending point were at the same location. The trajectories of the four groups were 65, 92.5, 9, and 25 m, and the walking speed was about 0.3–0.5 m/s.

**Chart 1:**
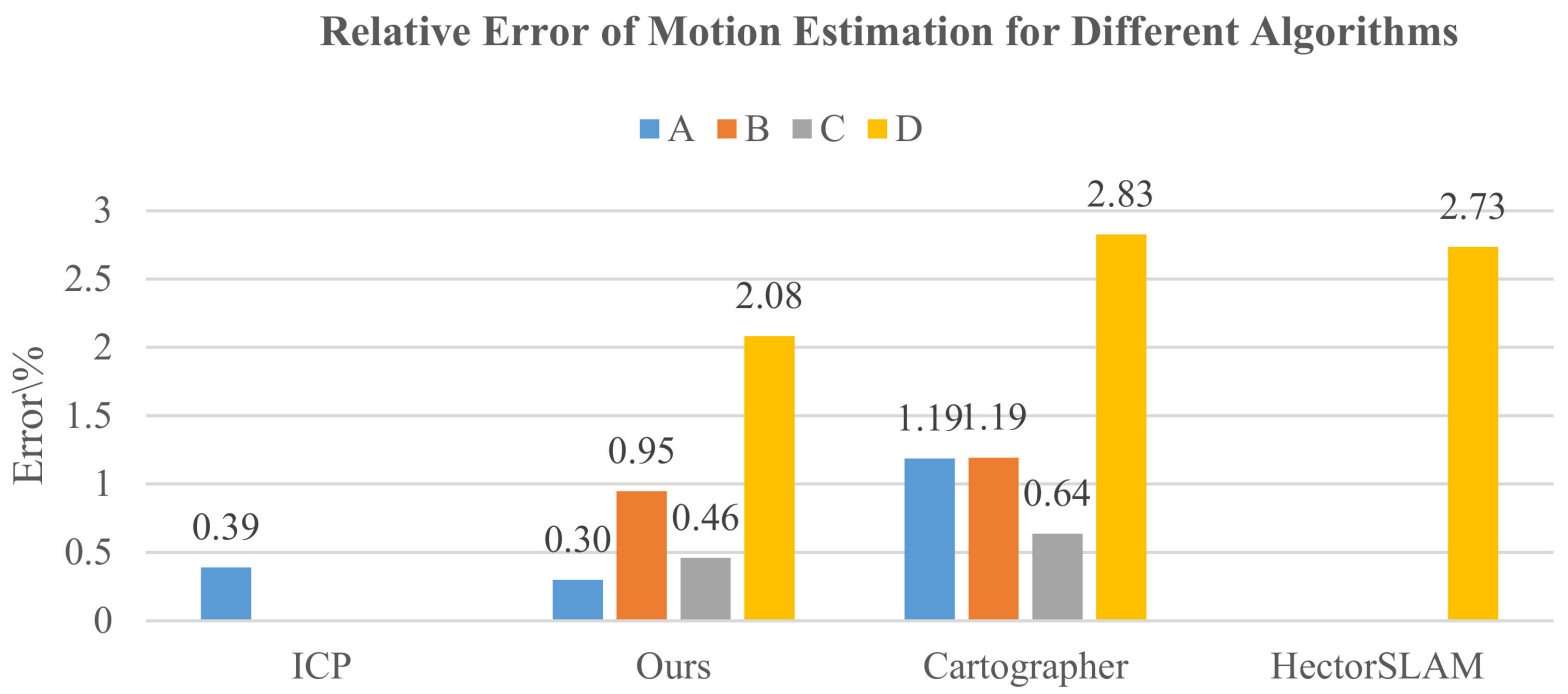
Relative errors of motion estimation drift for different algorithms. A, B, C represent the datasets of different scenes in the garage. D is the dataset of lab.

**Figure 6:**
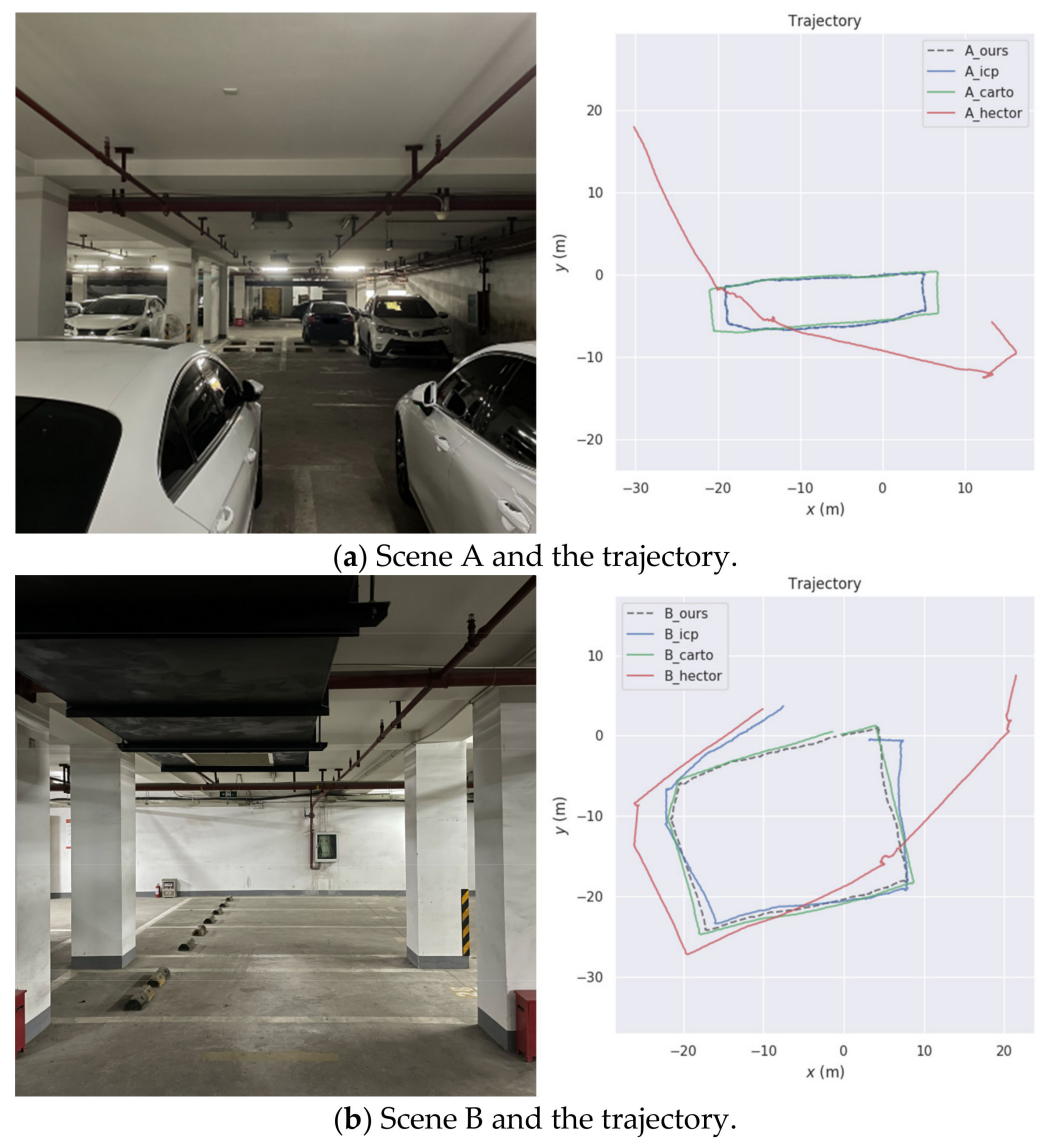

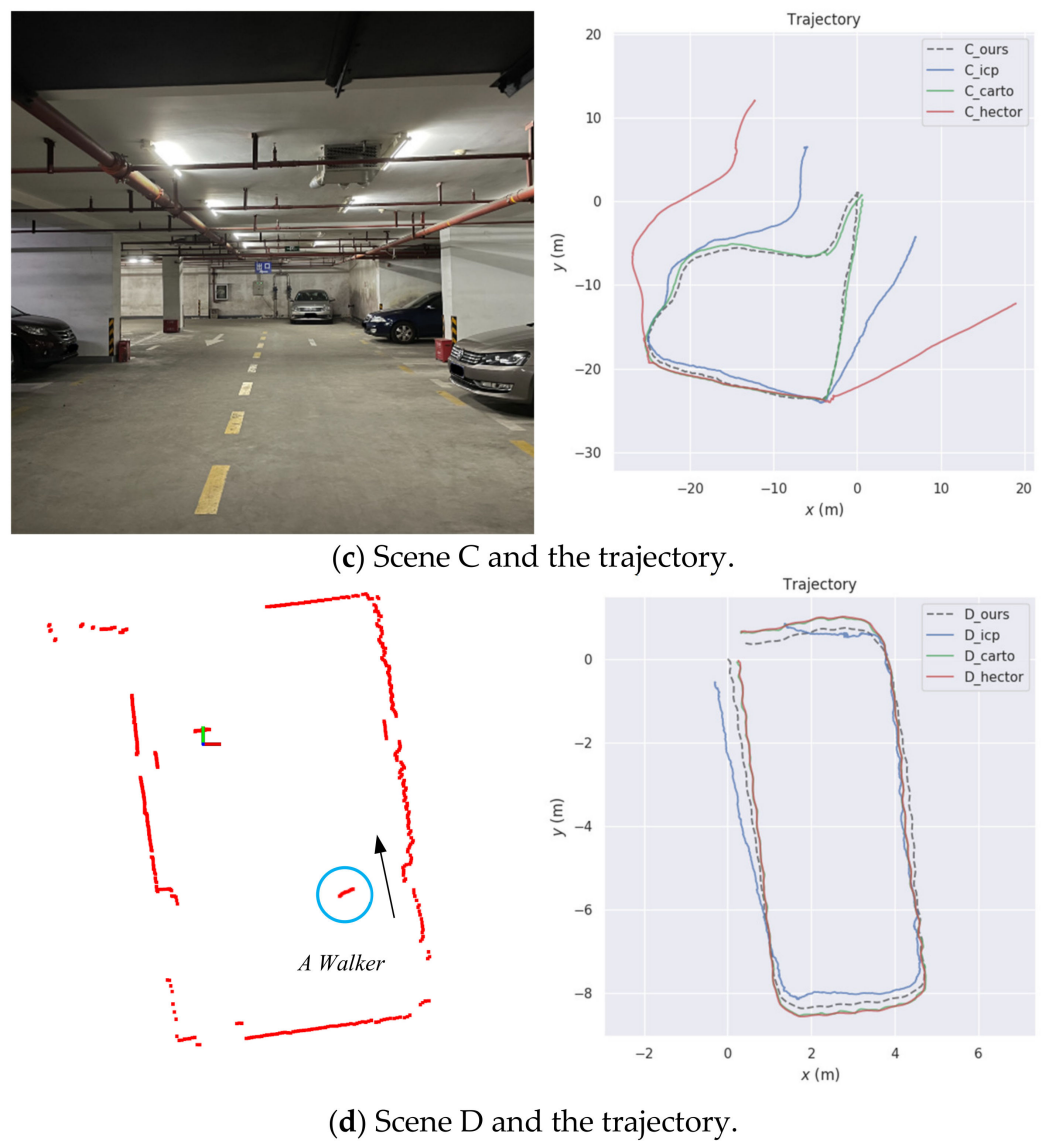
(**a**–**c**) Static scenes from the underground garage. (**d**) The dynamic scene from the lab.

We recorded the starting and ending positions and calculated the distance between the two positions, representing the displacement’s total drift. Chart 1 shows the four algorithm results, including the ICP, Cartographer, HectorSLAM, and our method. In general, the smaller the loop, the smaller the accumulated error. Our method outperformed the others. We omitted some data with an error of more than 10%. As shown in the trajectory in Figure 6 drawn by EVO [36], ICP and HectorSLAM did not perform very well in some datasets B, C, D and A, B, C, respectively. The HectorSLAM usually failed in rotation while our method performed well. Specifically, in scene D of Figure 6d, we collected the data when people were walking around in the lab and found that in general, the algorithms performed better in the static environment than in the dynamic environment but the error of our method was about 2%, and our method performed better than others.

To verify the real-time performance, the wall clock time of our method is recorded in Table 1, and the algorithm can achieve up to 2.9 times the real-time performance, and the minimum is 1.1 times. It can be noticed that the real-time performance drops in datasets E and F, mainly because the scanning frequency of the lidar used in these two data sets was 40 Hz, which is four times that of our lidar. This means that more data needs to be processed in the same period of time. In Table 2, we compute the runtime of the two main modules of our method in dataset B. For the large-scale feature extraction and data association, the average time per frame is about 56 ms; for the small-scale feature extraction and data association, the average time is about 26 ms. As one scan of RPLIDAR S1 takes 100 ms, our algorithm achieves real-time performance. We can also figure out that a small-scale feature extraction module takes less time than a large-scale feature extraction module due to the multi-scale data structure.

### 5.2. Open-Access Datasets

**Chart 2:**
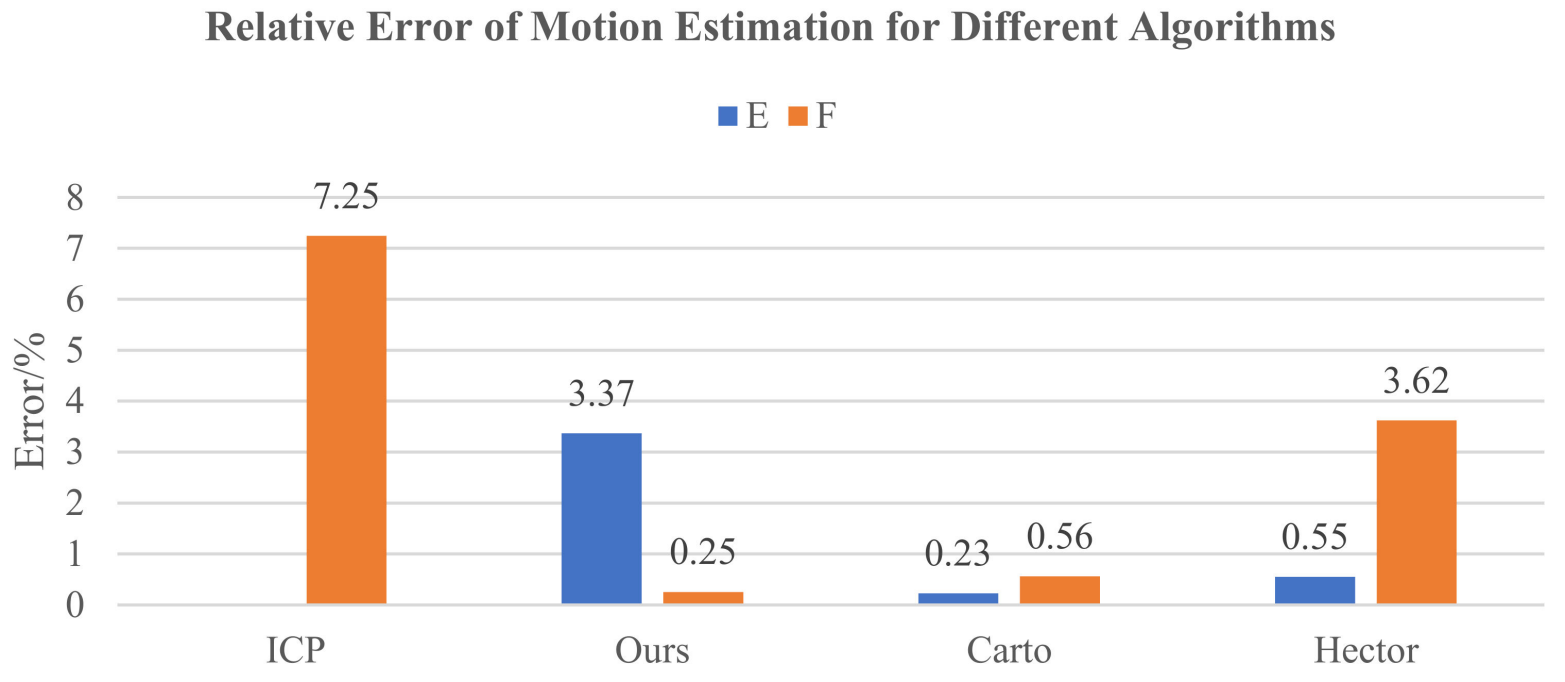
Relative errors of motion estimation drift for different algorithms. E and F are different open-access datasets.

We used the open-access datasets in [8]. To verify the low drift of the algorithm in this paper, we selected a large loop dataset located in the building of Schloss Dagstuhl and another with a small loop in a long and narrow corridor. The displacement’s total drift is shown in Chart 2, and our method outperformed Cartographer and HectorSLAM in dataset F. We omitted the data with an error of more than 10%.

**Figure 7:**
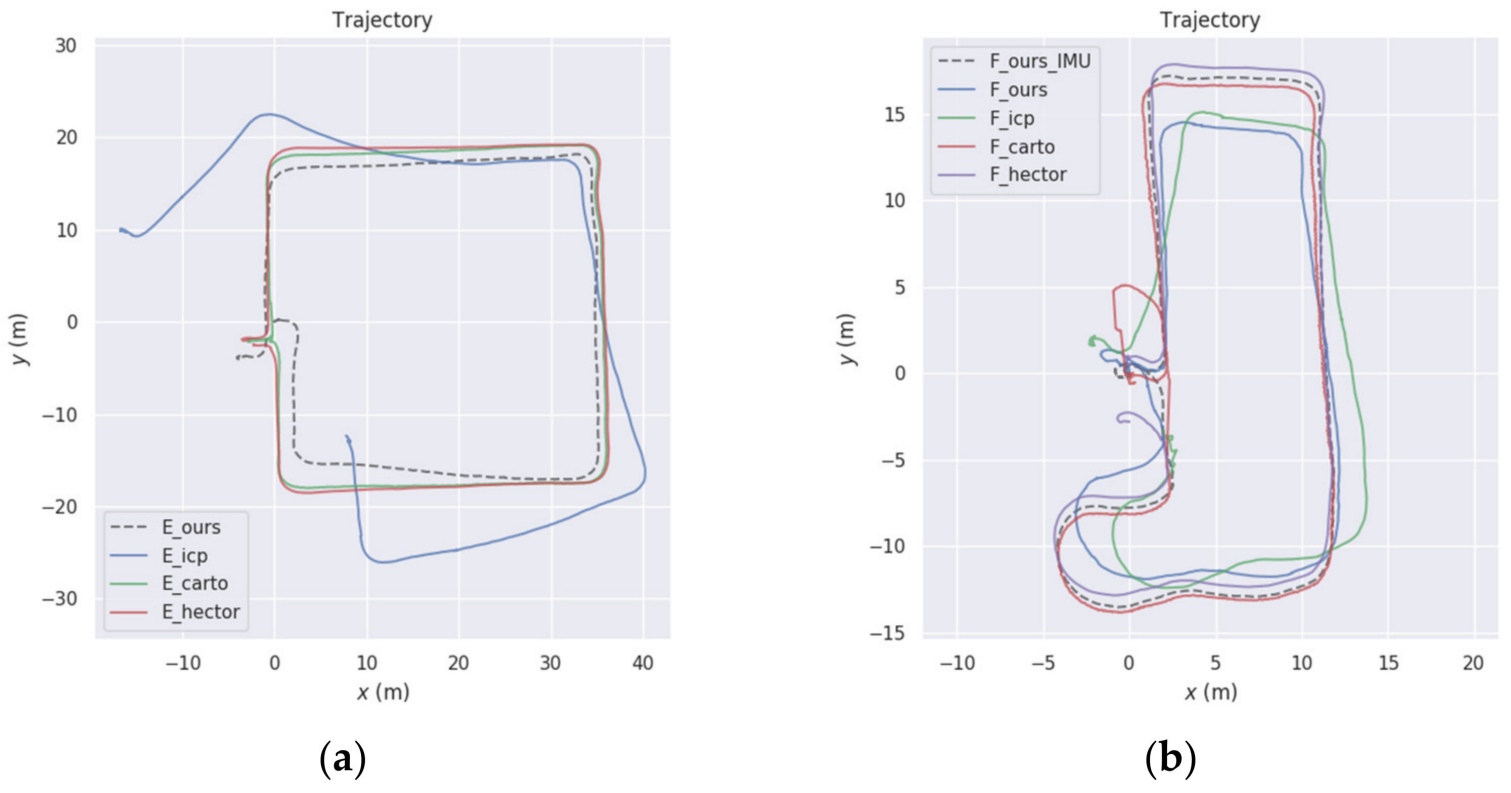
Trajectories for datasets E and F. (**a**) The trajectory of scene E, (**b**) The trajectory of scene F.

In dataset F, we regarded the wheel odometry data as ground truth and used EVO to calculate the absolute pose error (APE) and relative pose error (RPE). In Table 3, we learn that our method’s maximum absolute error of trajectory is 3.27 m, and the root mean square error (RMSE) is 1.71 m, the maximum relative error of trajectory is 0.19 m, and RMSE is 0.03. Few features can be extracted from the lidar data for the long and narrow corridor in dataset F. Therefore, we could find more displacement drift in the y-direction of Figure 7b. Since dataset F has IMU information, we first tried to apply IMU to remove the distortion of the point cloud with the reading of the accelerometer and gyroscope. Then, we used translation provided by IMU to assist our method to overcome the displacement drift in the long and narrow corridor. In Figure 7b, our method with IMU performed better than that without IMU.

## 6. Conclusions

This paper presents a real-time and low-drift 2-D lidar odometry algorithm for the indoor environment. We proposed a multi-scale metric procedure to extract robust features to utilize the 2-D lidar cloud information at multiple levels. Furthermore, the small-scale feature extraction component is less time-consuming due to omitting the secondary searching process. The proposed tangent-vector pair achieves robust performance when evaluating the rotation of the motion. The algorithm was validated on different datasets and compared with outstanding open-source algorithms. The results showed that our method can achieve better accuracy.

In future work, we intend to carry out research on multi-sensor fusion localization and mapping based on IMU and add a loop closure module to further improve the accuracy of the system.

## Figures and Tables

**Table 1:** Real-time performance of our method.

Dataset	Time of Bag/s	Wall Clock Time/s	Real-Time
A	192.5	67.2	2.9
B	245.3	145.4	1.7
C	134.3	75.1	1.8
D	81.9	47.9	1.7
E	145.4	129.2	1.1
F	105.2	89.6	1.2

**Table 2:** Runtime of the two main modules.

Module	Max (ms)	Min (ms)	Mean (ms)
Large-scale feature and data association	89.3	10.7	56.1
Small-scale feature and data association	64.9	5.1	25.9

**Table 3:** Absolute pose error and relative pose error.

Dataset	Error	Max/m	Min/m	Mean/m	RMSE/m
F	APE	3.27	0.01	1.42	1.71
	RPE	0.19	<0.01	0.02	0.03
